# Modification of American Joint Committee on cancer prognostic groups for renal cell carcinoma

**DOI:** 10.1002/cam4.1790

**Published:** 2018-10-10

**Authors:** Ning Shao, Hong‐Kai Wang, Yao Zhu, Ding‐Wei Ye

**Affiliations:** ^1^ Department of Urology Fudan University Shanghai Cancer Center Shanghai China; ^2^ Department of Oncology, Shanghai Medical College Fudan University Shanghai China

**Keywords:** AJCC staging system, overall survival, renal cell carcinoma

## Abstract

**Background:**

To compare the predictive value of the current AJCC stage grouping for renal cell carcinoma (RCC) to our modifications.

**Patients and methods:**

A total of 2120 patients with RCC from Fudan University Shanghai Cancer Center (FUSCC) database and 74 506 counterparts from SEER database were included. Cox regression was used to calculate the relative impacts between prognostic groups. The predictive accuracy of overall survival (OS) was assessed using the concordance index (C‐index), which was compared by likelihood ratio test.

**Results:**

In FUSCC cohort, the 5‐year‐OS rate for T3N0M0 patients was higher than T1‐3N1M0 (72.7% vs 38.1%). The 5‐year‐OS rate for T4N0M0 was 36.2%, which was close to T1‐3N1M0 but not to T4N1M0 (0%) and TanyNanyM1 (12.6%). The elements of AJCC groups were regrouped according to the ranks of hazard ratios. The modified stages II (T3N0M0), III (T1‐3N1M0, T4N0M0), and IV (T4N1M0, TanyNanyM1) exhibited greater survival stratification than AJCC groups. The modifications were validated in SEER cohort and yielded similar survival outcomes. The predictive accuracy of OS in modified prognostic groups was significantly higher than AJCC groups in stages II‐IV subgroups in both FUSCC (C‐index: 0.801 vs 0.779, *P* < 0.001) and SEER cohort (C‐index: 0.770 vs 0.764, *P* < 0.001).

**Conclusions:**

The modified AJCC prognostic groups for RCC provided significantly improved survival prediction compared with the 8th AJCC edition. A precise risk stratification of modified stages II‐IV disease provides an important basis for risk‐equivalent treatment recommendation.

## 1 INTRODUCTION

Nearly 63 990 new cases of kidney and renal pelvis cancer were expected to be diagnosed in the USA in 2017, accounting for about 5% of all new cases in males and 3% in females.^1^, ^2^ Tumor stage is considered as the most important prognostic parameter for the clinical behavior and outcome of renal cell carcinoma (RCC).^3^

The American Joint Committee on Cancer (AJCC) tumor‐node‐metastasis stage grouping (TNM) is the most commonly used cancer staging system.^4^, ^5^, ^6^ The eighth edition (8th) of the AJCC stage grouping was the latest version. Despite some minor revisions in comparison with the seventh (7th) system, there were no changes in the AJCC prognostic stage grouping. It means that the system of old prognostic stage grouping has no changes in nearly ten years and will continue to be used.

Stage grouping (TNM) plays an important role in treatments decisions according to the National Comprehensive Cancer Network (NCCN) guidelines, especially in the selection criteria for adjuvant therapy.^7^ High‐risk patients with RCC were recommended for clinical trials and adjuvant therapy. Therefore, precise stage grouping is critical for accurate risk stratification in RCC.^8^ However, problems arose in the selection of suitable patients for adjuvant therapy with the application of AJCC stage grouping in various populations.^9^, ^10^, ^11^

Given the need for more precise stage grouping and treatment stratification,^12^ we investigated the overall survival (OS) of each subgroup and refined prognostic stage grouping in a large population of patients. Hence, the purpose of our study was to validate the predictive value and feasibility of our modifications in AJCC prognostic stage grouping.

## 2 METHODS

### 2.1 Patients

#### 2.1.1 FUSCC cohort

The Fudan University Shanghai Cancer Center (FUSCC) cohort group (training set) of patients with RCC was obtained from the FUSCC (2000‐2015). Our study was approved by the Ethics Committee of FUSCC. All patients with RCC have been histologically confirmed by surgery or biopsy in our department. In addition, abdominal/pelvic computed tomography (CT) scan and Magnetic Resonance Imaging (MRI) were used when needed. Patients included in this study were staged according to the definitions of the 8th AJCC stage grouping. After informed consent was obtained, patients were well informed of the importance of follow‐up. Patients were regularly followed up every 3 months for the first 3 years, then every 6 months up to 5 years, then annually thereafter.

#### 2.1.2 SEER cohort

The Surveillance, Epidemiology, and End Results (SEER) cohort was used as the test set because of its large sample size. From the SEER database, the test cohort data were retrieved from 2004 to 2014. Only patients with microscopically confirmed RCC (using ICD‐O‐3 histology/behavior codes: 8260/3, 8270/3, 8290/3, 8310/3, 8312/3, 8316/3, 8317/3, 8319/3, 8320/3, 8323/3, 8480/3, and 8510/3) were included. The other variables such as year of diagnosis, age at diagnosis, race/ethnicity, and sex were also collected. For staging information, the following codes were obtained from SEER: (a) Derived AJCC Stage Group, 6th ed (2004+), Derived AJCC Stage Group, 7th ed (2010+). (b) Derived AJCC T, 6th ed (2004+), Derived AJCC T, 7th ed (2010+). (c) Derived AJCC N, 6th ed (2004+), Derived AJCC N, 7th ed (2010+). (d) Derived AJCC M, 6th ed (2004+), Derived AJCC M, 7th ed (2010+). (e) OS and follow‐up data. In addition, TxNanyM0, TxNanyMx, TanyNxM0, and TanyNxMx patients were excluded. If patients had both 6th and 7th AJCC Stage Group information, they were restaged according to the definitions of TNM the 7th AJCC staging system. Patients with tumor that directly invaded the ipsilateral adrenal gland were classified into T3a in 6th AJCC Stage Group but T4 in 7th AJCC Stage Group. The patients classified into T3a only by 6th AJCC Stage System information were excluded. Flow diagram of selection is outlined in Figure S1.

### 2.2 Statistical analyses

OS is defined as the months from the date of diagnosis to the date of death or last follow‐up. Patients were censored if they were lost to follow‐up or died. To assess the associations between each group in stage grouping and outcome, Kaplan‐Meier curves and log‐rank tests were used. Cox proportional hazards analysis was used to assess the relative impacts of different stages on OS.

The concordance index (C‐index) and heatmaps were performed to evaluate the discriminatory powers of the two staging systems. To assess predictive ability, likelihood ratio test was used to compare the C‐index of both staging system. All statistical analyses were performed using R (version 3.4.2, http://www.r-project.org). All statistical tests were 2‐sided, and *P* value < 0.05 was considered statistically significant.

## 3 RESULTS

### 3.1 Cohort characteristics

FUSCC cohort of 2120 RCC patients with 68.4% of males were included. The median age of the patients at diagnosis was 55.0 years. Additionally, 74 506 patients from SEER cohort were included. Of these, 46 928 (63%) were male and 27 578 (37%) were female. The median age at diagnosis was 60.3 years. Table 1 summarizes the demographic and clinical characteristics of the patients in both cohort.

**Table 1:** The demographic and clinical characteristics of SEER and FUSCC cohort

	SEER cohort	FUSCC cohort
Characteristics	n = 74 506	n = 2120
Age, years		
<65	45 933 (61.65)	1633 (77.03)
≥ 65	28 573 (38.35)	487 (22.97)
Sex		
Male	46 928 (62.99)	1450 (68.40)
Female	27 578 (37.01)	670 (31.60)
8th AJCC TNM stage		
T1N0M0	47 954 (64.36)	1493 (70.42)
T1N1M0	122 (0.16)	19 (0.90)
T2N0M0	7753 (10.41)	190 (8.96)
T2N1M0	192 (0.26)	19 (0.90)
T3N0M0	7162 (9.61)	164 (7.74)
T3N1M0	531 (0.71)	31 (1.46)
T4N0M0	325 (0.44)	16 (0.75)
T4N1M0	85 (0.11)	9 (0.42)
TanyNanyM1	10 382 (13.93)	179 (8.44)
8th AJCC prognostic stage		
I	47 954 (64.36)	1493 (70.42)
II	7753 (10.41)	190 (8.96)
III	8007 (10.75)	233 (10.99)
IV	10 792 (14.48)	204 (9.62)
Modified 8th AJCC prognostic stage		
I	55 707 (74.77)	1683 (79.39)
II	7162 (9.61)	164 (7.74)
III	1170 (1.57)	85 (4.01)
IV	10 467 (14.05)	188 (8.87)
Histopathologic type		
Clear cell renal cell carcinoma	43 959 (59.00)	1740 (82.08)
Papillary renal cell carcinoma	8644 (11.60)	89 (4.20)
Chromophobe renal cell carcinoma	4160 (5.58)	82 (3.87)
Collecting duct renal cell carcinoma	201 (0.27)	6 (0.28)
Renal medullary carcinoma	48 (0.06)	15 (0.71)
Other renal cell carcinoma	17 494 (23.48)	188 (8.87)

AJCC, American Joint Committee on Cancer; FUSCC, Fudan University Shanghai Cancer Center; SEER, Surveillance, epidemiology, and end results.

Data are presented as number (percentage) unless otherwise indicated.

### 3.2 Modification of the 8th AJCC Staging System based on OS

**Figure 1:**
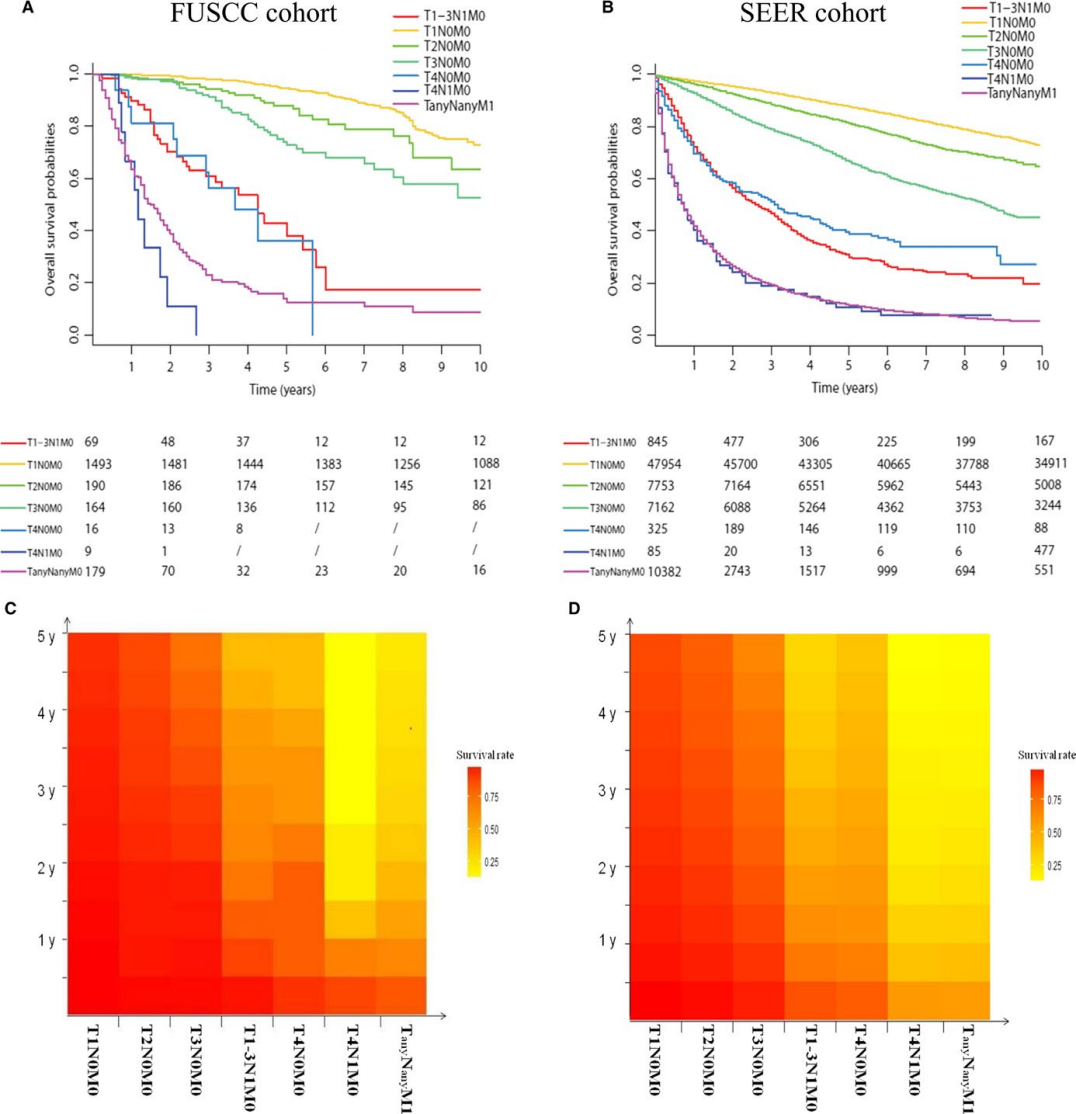
Kaplan‐Meier survival curves of the patients in T1‐3N1M0, T1N0M0, T2N0M0, T3N0M0, T4N0M0, T4N1M0, and TanyNanyM1 from (A) the FUSCC cohort and (B) the SEER cohort. Color variation on the *Y* axis of the heatmaps reflected the 5‐year‐OS rates variation of these patients from (C) the FUSCC cohort and (D) the SEER cohort

The predictive value of the 8th AJCC staging system for RCC in the Chinese cohort using Kaplan‐Meier survival analysis based on the splitting of the TNM subgroups (T1N0M0, T1N1M0, T2N0M0, T2N1M0, T3N0M0, T3N1M0, T4N0M0, T4N1M0, TanyNanyM1) was performed in the FUSCC cohort. Figure S2A shows that the OS of the subgroup of T3N0M0 (stage III) and T4N0M0 (stage IV) were much better than the other subgroups in the same 8th AJCC stage grouping, respectively (stage III: T1N1M0, T2N1M0, and T3N1M0; stage IV: T4N1M0 and TanyNanyM1). The number of patients in T1N1M0, T2N1M0, or T3N1M0 was so small in our cohort, and these patients were amalgamated into one subgroup (T1‐3N1M0). Furthermore, the survival curve of T4N0M0 was very close to the curve of T1‐3N1M0, while the survival curve of T3N0M0 was much higher than that of T1‐3N1M0 (Figure 1A). In addition, the 5‐year‐OS rate for T3N0M0 was significantly higher than T1‐3N1M0 (72.7% vs 38.1%). The 5‐year‐OS rate for T4N0M0 was 36.2% (Table S1), which was close to T1‐3N1M0 but not to T4N1M0 (0%) and TanyNanyM1 (12.6%).

**Figure 2:**
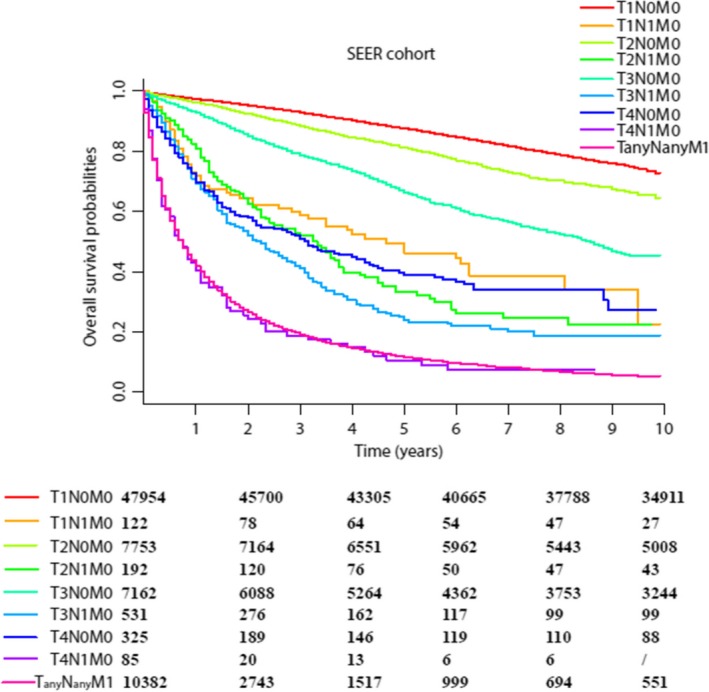
(A) Kaplan‐Meier survival curves for the patients of different TNM subgroup from the SEER cohort. (B) The 8th Editions of the AJCC Staging Definitions and the Modified 8th Staging Definitions for RCC

Hence, the current 8th AJCC staging system may not be accurate and appropriate. We regrouped the AJCC prognostic stage grouping according to the OS of each subgroup without changing the definition of TNM. T1N0M0 and T2N0M0 were classified as stage I, and this was subdivided into IA (T1N0M0) and IB (T1N0M0). T3N0M0 was classified as stage II. T1‐3N1M0 and T4N0M0 were classified as stage III. T4N1M0 and TanyNanyM1 were classified as stage IV (Figure 2B).

The modified AJCC stage grouping was better suitable for the outcomes of patients. Similar results were obtained for the hazard ratios (HRs) compared with T1N0M0 disease. The HRs of T4N0M0 (13.07) were closer to T1‐3N1M0 (12.46) rather than T4N1M0 (69.14) and TanyNanyM1 (29.16). Additionally, the HRs of T3N0M0 (3.21) were significantly lower than T1‐3N1M0 (Table 2).

**Table 2:** HRs of different staging group and C‐indexes in SEER and FUSCC cohort

Staging system		SEER cohort	FUSCC cohort
8th AJCC staging system		HR (95% CI)	HR 95% CI
Stage I	T1N0M0	/	/
Stage II	T2N0M0	1.53 (1.44‐1.63)	1.89 (1.29‐2.77)
Stage III	T1‐3N1M0 + T3N0M0	3.23 (3.08‐3.40)	4.82 (3.64‐6.39)
Stage IV	TanyNanyM1+T4N0‐1M0	18.36 (17.73‐19.03)	27.66 (21.77‐35.14)
Modified 8th AJCC staging system			
Stage Ia	T1N0M0	/	/
Stage Ib	T2N0M0	1.53 (1.44‐1.63)	1.89 (1.29‐2.77)
Stage II	T3N0M0	2.76 (2.61‐2.92)	3.21 (2.27‐4.55)
Stage III	T1‐3N1M0 + T4N0M0	7.80 (7.19‐8.46)	12.82 (9.08‐18.10)
Stage IV	TanyNanyM1 + T4N1M0	19.11 (18.44‐19.80)	30.02 (23.54‐38.27)
	T1N0M0	/	/
	T2N0M0	1.53 (1.44‐1.63)	1.89 (1.29‐2.77)
	T3N0M0	2.76 (2.61‐2.92)	3.21 (2.27‐4.55)
	T1N1M0	5.68 (4.41‐7.33)	8.29 (4.05‐16.96)
	T2N1M0	7.15 (5.93‐8.62)	17.53 (9.67‐31.78)
	T3N1M0	9.44 (8.41‐10.60)	12.93 (7.52‐22.23)
	T1‐3N1M0	8.14 (7.41‐8.94)	12.46 (8.54‐18.19)
	T4N0M0	6.96 (5.97‐8.12)	13.07 (6.84‐24.98)
	T4N1M0	18.84 (14.95‐23.74)	69.14 (34.56‐138.33)
	TanyNanyM1	19.12 (18.46‐19.82)	29.16 (22.81‐37.29)
C‐indexes (stages II and IV patients)			
8th AJCC staging system		0.764 (0.758‐0.769)	0.779 (0.743‐0.815)
Modified 8th AJCC staging system		0.770 (0.765‐0.776)	0.801 (0.765‐0.838)

CI, confidence interval; C‐index, concordance index; HRs, hazard ratios compared with T1N0M0.

### 3.3 Verification of modified 8th AJCC Staging System based on SEER cohort

The trends of survival curve in each subgroup in SEER cohort strengthened the need and appropriateness for the modification of stage grouping. As shown in Figure 1B, T4N0M0 and T1‐3N1M0 were more suitable in one stage than T3N0M0. Besides, each subgroup of T1‐3N1M0 (such as T1N1M0, T2N1M0, and T3N1M0) showed worsened prognostic rates significantly than T3N0M0 (Figure 2A).

As the patients in T1‐3N1M0 were far more than the FUSCC cohort, the improved changes in this classification were more visible and reliable. The 5‐year‐OS rates for T3N0M0, T1‐3N1M0, and T4N0M0 patients were 66.4%, 30.0%, and 39.0%, respectively (Table S1). The HRs of T4N0M0, T1‐3N1M0, and T3N0M0 compared with T1N0M0 were 6.96, 8.14, and 2.76, respectively (Table 2). We found that T1‐3N1M0 and T3N0M0 were arranged in the same stage III (HR = 3.23) in the 8th AJCC stage grouping was unreasonable. Besides, the HRs of T4N0M0 were much lower than T4N1M0 (18.84) and TanyNanyM1 (19.12).

**Figure 3:**
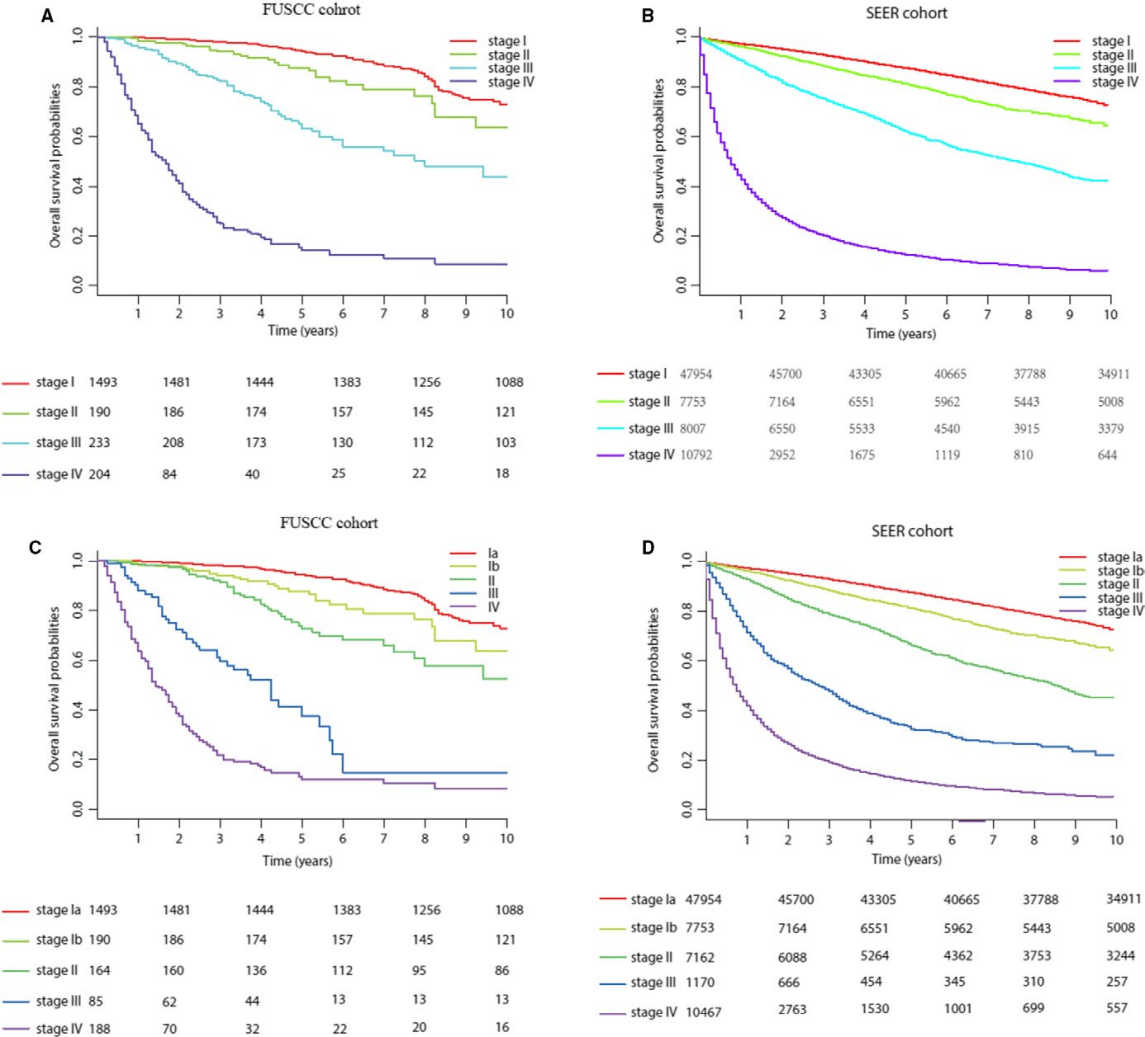
Kaplan‐Meier survival curves of the patients from (A) the FUSCC cohort and (B) the SEER cohort according to the 8th AJCC staging system. Kaplan‐Meier survival curves for the patients from (C) the FUSCC cohort and (D) the SEER cohort according to the modified 8th staging system

The heatmaps (Figure 1C,D) reflected the modified stage more intuitively. The subgroup with similar color variation on the Y axis should be assigned to the same stage. It was not hard to find that the modified stage grouping was more in line with color variation than the 8th AJCC stage grouping. As the huge number of T1N0M0 patients could diminish the better discrimination in stages II‐IV patients, C‐indexes of two types of staging systems were only calculated for patients in stages II‐IV (Table 2). C‐indexes for the modified 8th stage grouping were improved significantly (0.770, 95% CI: 0.765‐0.776 vs 0.764, 95% CI: 0.758‐0.769) than the 8th stage grouping in SEER cohort. The *P*‐value of likelihood ratio test was <0.001. Similar results could be found in the FUSCC cohort. Based on the above analysis, our results validated significant improvements of the modified 8th AJCC stage grouping. (Figure 3)

### 3.4 Sensitivity analysis

Because the SEER cohort was much larger than the FUSCC cohort, especially in the T1‐3N1M0 group, we used SEER cohort to conduct a sensitivity analysis. To identify different independent variables that may impact our modified stage grouping, stratified analysis was performed based on the histopathological types (ccRCC, papillary RCC, chromophobe carcinoma, and other RCC), race, and years at diagnosis (2004‐2010, after 2010). As shown in Figure S3, both ccRCC and non‐ccRCC (including papillary RCC, chromophobe carcinoma, and other RCC) demonstrated similar results. The OS of T3N0M0 was much better than T1‐3N1M0, and T4N0M0 was more suitable in the same stage with T1‐3N1M0. Then, according to the race recode variables (White, Black, Other) in SEER database, KM‐survival analysis was also performed (Figure S2B‐D). The results indicated that the outcome of T3N0M0 and T4N0M0 was much better than other subgroups in the same stage. Finally, the patients with diagnosis years between 2004 and 2010 and after 2010 also showed similar trends (Figures S3 and S4), meaning that our modified 8th AJCC stage grouping was feasible and credible.

## 4 DISCUSSION

The most important function of stage grouping is to predict outcomes as accurately as possible.^13^ Interestingly, our results indicated that 8th AJCC Staging System had much room for improvement. This is because both stages III and IV included two types of subgroups with significantly different prognosis. T3N0M0, with much better outcome, was classified into stage III with T1‐3N1M0. Similarly, T4N0M0 was classified into stage IV inappropriately with T4N1M0 and TanyNanyM1. These findings suggested that 8th AJCC stage grouping underestimated the prognosis of T3or4N0M0. Indeed, appropriate and minor modifications of AJCC stage grouping that can bring more precise prediction of prognosis are necessary.^14^, ^15^

Our modifications without changing the definition of TNM may be considered as a good choice. The proposed system furthermore partitions risk over a great spectrum: patients in stages II, III, and IV in the proposed system have 2.76, 7.80, and 19.11 times risk of death, respectively, compared with T1N0M0 patients. In comparison, the HR for 8th AJCC stages II, III, and IV patients is 1.53, 3.23, and 18.36, respectively. Approximately 10% RCC patients (T3N0M0: 9.6%, T4N0M0: 0.4% according to SEER cohort) were regrouped according to our modifications, which made the survival curves separated accurately between the stages. The greatest impact of this modifications is not the subtle and appropriate reclassification, but in the characterization of real high‐risk subgroup.

The other important function of the AJCC stage grouping is to help clinical treatment decision‐making process, evaluate treatment efficacy, and determine the selection criteria for clinical trials.^16^, ^17^ As TNM stage grouping is the most widely used staging system, it also reflects the treatment changing paradigm.^18^, ^19^ The definition of stage Ia and Ib reflects better outcome of both T1N0M0 and T2N0M0, which means a good outcome for these patients in nowadays management. Similarly, the upgrading of T3N0M0 and T4N0M0 indicates that surgical consolidation in localized massive disease is more feasible in current surgical treatments. Only high‐risk patients with ccRCC are recommended for the use of adjuvant therapy (Sunitinib) as an option according to the NCCN guidelines unlike others in stages II and III.^7^ The intension of adjuvant trials was to delay or diminish recurrence in high‐risk patients. However, there were two famous randomized controlled trials (RCTs) with contrary results. The HR of S‐TRAC RCT was 0.76 (95% CI: 0.59‐0.98) with 90.6% and 90.8% T3N0M0 patients in Sunitinib and Placebo groups, respectively.^20^ The HR (AJCC stages III‐IV) of ASSURE RCT was 1.01 (95% CI: 0.81‐1.25) with 97.9% and 99.0% stage III patients (there was no details data in stage III) in Sunitinib and Placebo groups, respectively.^21^ The 5‐year‐OS rates were nearly 60% in Sunitinib and 46% in Placebo in high‐risk patients in S‐TRAC RCT. The 5‐year‐OS rates were nearly 51% in both Sunitinib and Placebo groups in high‐risk patients in ASSURE RCT. Our results showed that the 5‐year‐OS rates of T3N0M0 patients were 66.4% in SEER cohort and 72.7% in FUSCC cohort, both of which were much higher than that of patients in the placebo/Sunitinib in RCTs. It indicated that T3N0M0 may not be considered as high‐risk stage as considered before. These patients may not benefit from the adjuvant therapy significantly. Importantly, our modified stage grouping emphasizes the better prognostic of T3N0M0 patients and suggests that these patients should not be treated as equivalent to T1‐3N1M0 patients. Given the poor outcomes in stages III and IV patients in our modified stage grouping, these patients may derive great benefit from adjuvant treatments. Additionally, they may also be excellent candidates for novel treatments, such as immunotherapy. Besides, overestimating the risks of T3N0M0 (9.6% of RCC patients) implied a higher disease burden with overtreatment of adjuvant therapy.^22^, ^23^, ^24^ Evaluation of the efficacy and excessive treatment could be solved better if the future adjuvant therapy RCTs adopted the modified AJCC staging system and performed subgroup analysis accordingly. Collectively, our modified AJCC staging system may have some impact on the adjuvant therapy trials setting which is highly controversial according to recent trials.

The strengths of this study were larger sample size, an adequate number of death events, external validation in SEER database, and reproducible test. Additionally, modifications were only in staging group without changing the definition of TNM. There are also some limitations to our study. Firstly, the study was limited by its retrospective nature. The SEER database may still have the possibility of coding errors or erroneous data. Secondly, FUSCC cohort and SEER cohort did not reach all the people. Hence, future studies with larger number of participants from worldwide could validate our conclusions.

## 5 CONCLUSIONS

The present large study suggested that the current 8th AJCC stage grouping for RCC had much room for improvement. According to the OS of each subgroup, we mainly modified the AJCC prognostic stage groups, especially of stages III and IV. T3N0M0 was classified as an independent stage. T1‐3N1M0 and T4N0M0 were classified as stage III. T4N1M0 and TanyNanyM1 were classified as stage IV. This modified AJCC stage grouping is proved to be a more precise prediction of prognosis. Additionally, it is feasible and credible. Hence, the modification of 8th AJCC stage grouping brings new insights on the next version of the AJCC stage grouping and adjuvant therapies in the future RCTs.

## ETHICS APPROVAL AND CONSENT TO PARTICIPATE

Our study was approved by the Ethics Committee of FUSCC. Additionally, informed consent was obtained.

## CONFLICTS OF INTEREST

The authors declare no conflict of interest.
