# Supplementary material for: Modification of American Joint Committee on cancer prognostic groups for renal cell carcinoma
Source: Cancer Med. 2018 Oct 10;7(11):5431–8. doi: 10.1002/cam4.1790 (PMC6247054; doi:10.1002/cam4.1790)

Supplementary Figure Legends

Supplementary Figure S1 Flow diagram of selection in FUSCC and SEER cohort

Supplementary Figure S2 Kaplan-Meier survival curves of the patients in each subgroup of TNM in the FUSCC cohort (A). Kaplan-Meier survival curves of the patients according to the race recode variables [White (B), Black (C), Other (D)] in SEER database.

Supplementary Figure S3 Kaplan-Meier survival curves of the patients according to histopathological types [ccRCC (C), other RCC (D), papillary RCC (E) and chromophobe carcinoma (F)] and years at diagnosis [(2004-2010 (A), after 2010 (B)] in SEER database.

Supplementary Figure S4 Kaplan-Meier survival curves of the patients from the SEER cohort according to years at diagnosis [(2004-2010 (A,B), after 2010 (C,D)] in terms of 8th AJCC staging system or modified 8th staging system.

supplementary Figure S1

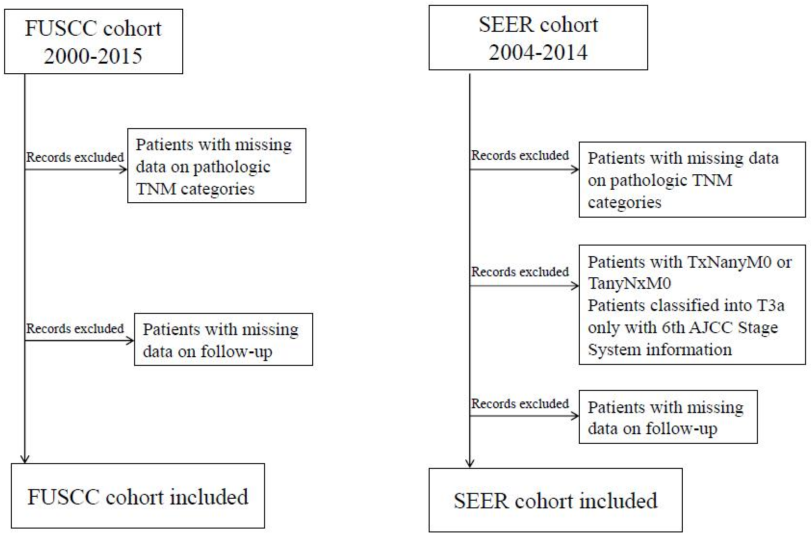

supplementary Figure S2

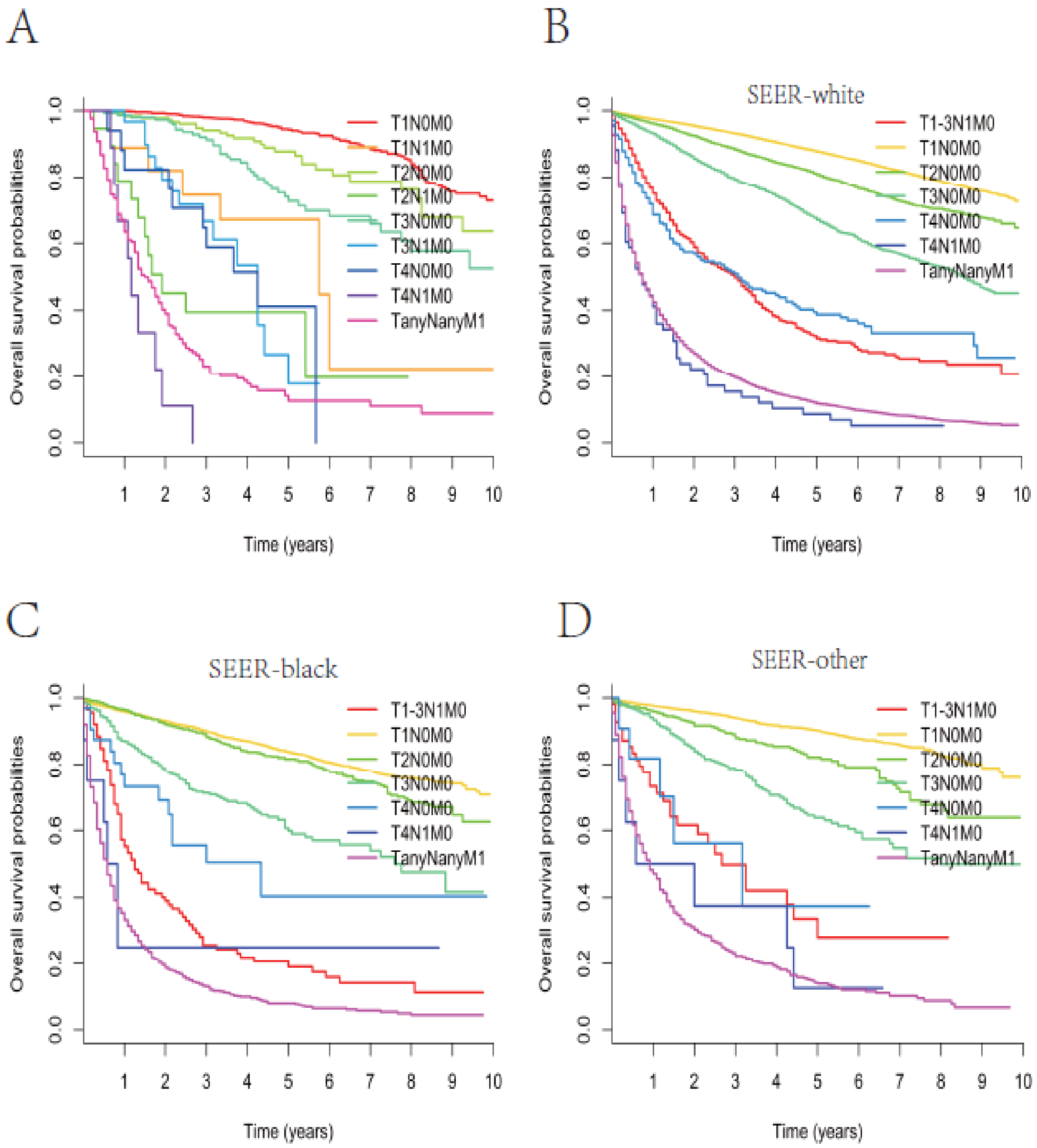

supplementary Figure S3

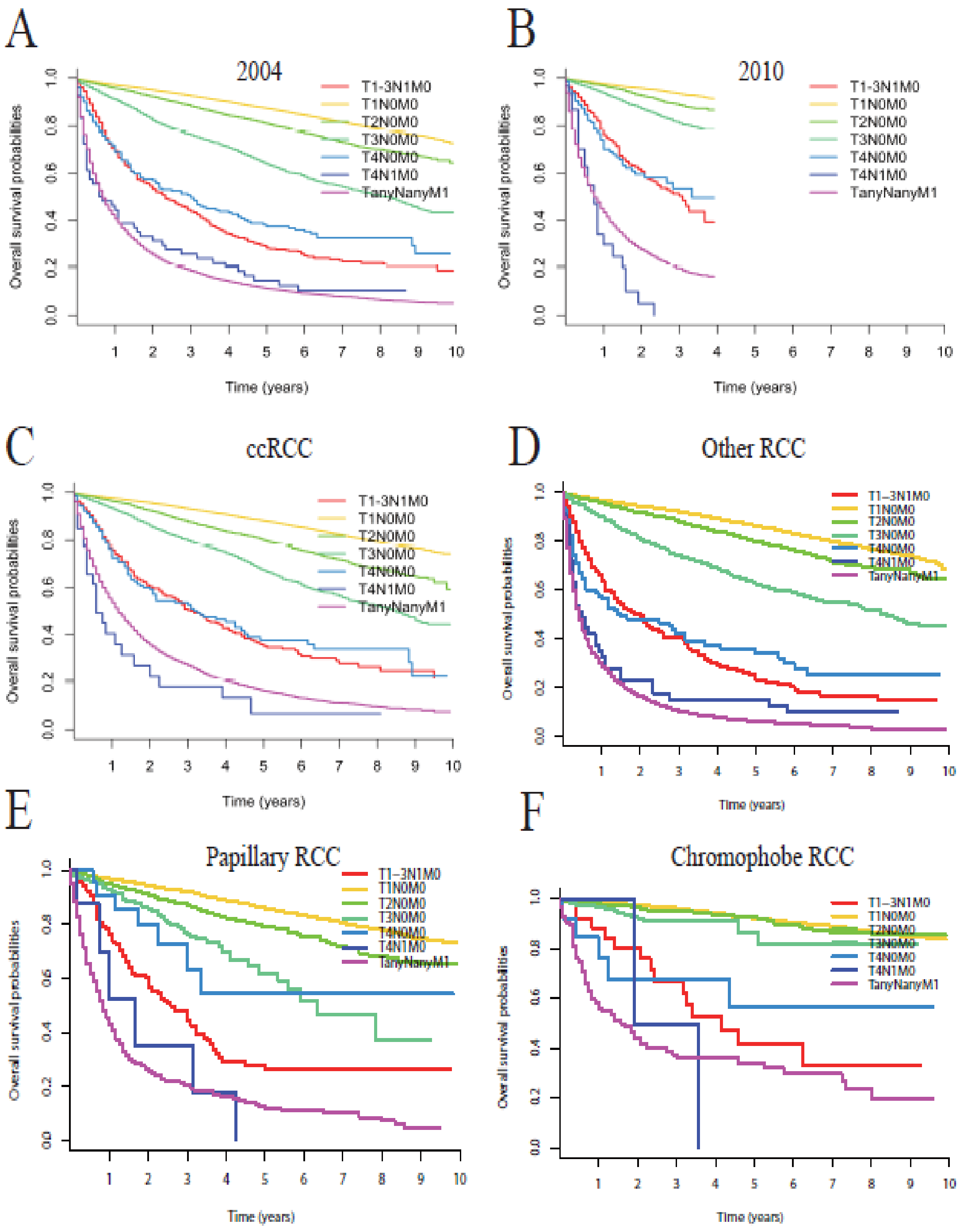

supplementary Figure S4

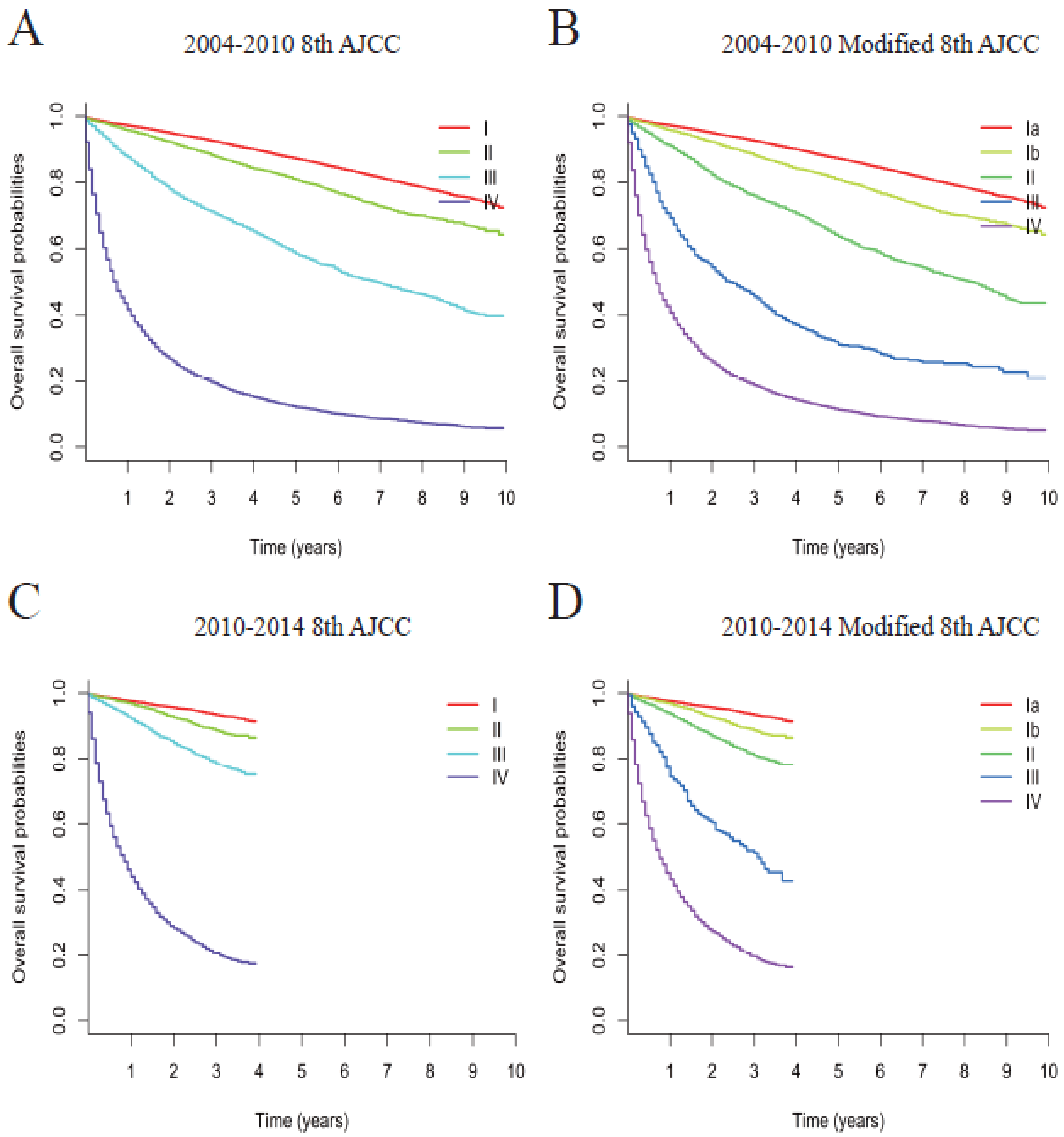

Supplement: Supplementary file 1 [file CAM4-7-5431-s001.pdf]
