# Supplementary material for: Modification of American Joint Committee on cancer prognostic groups for renal cell carcinoma
Source: Cancer Med. 2018 Oct 10;7(11):5431–8. doi: 10.1002/cam4.1790 (PMC6247054; doi:10.1002/cam4.1790)
Supplement: Supplementary file 2 [file CAM4-7-5431-s002.doc]

| Staging System |  | SEER cohort | | | | |  | FUSCC cohort | | | | |
| --- | --- | --- | --- | --- | --- | --- | --- | --- | --- | --- | --- | --- |
|  |  | 1y | 2y | 3y | 4y | 5y |  | 1y | 2y | 3y | 4y | 5y |
| T1N0M0 |  | 97.4% | 95.3% | 92.9% | 90.3% | 87.5% |  | 99.9% | 99.2% | 98.1% | 96.7% | 94.5% |
| T2N0M0 |  | 96.2% | 92.4% | 88.4% | 84.5% | 81.2% |  | 98.4% | 97.9% | 94.2% | 91.8% | 87.8% |
| T3N0M0 |  | 92.7% | 85.0% | 78.8% | 73.5% | 66.4% |  | 98.8% | 97.4% | 91.3% | 82.7% | 72.7% |
| T1-3N1M0 |  | 72.3% | 56.4% | 46.5% | 36.2% | 29.9% |  | 89.6% | 70.1% | 60.9% | 53.6% | 38.1% |
| T4N0M0 |  | 69.5% | 58.1% | 51.1% | 44.9% | 39.0% |  | 81.2% | 81.2% | 62.5% | 48.2% | 36.2% |
| T4N1M0 |  | 40.3% | 24.2% | 18.8% | 14.8% | 10.6% |  | 66.7% | 11.1% | 0% | 0% | 0% |
| TanyNanyM1 |  | 41.8% | 26.4% | 19.2% | 14.6% | 11.6% |  | 63.4% | 38.9% | 23.2% | 17.8% | 12.6% |

Appendix TableA1

The 1-5 year OS rates of different groups in SEER and FUSCC cohort

y: year
